# A comprehensive review of electrophysiological techniques in amyotrophic lateral sclerosis research

**DOI:** 10.3389/fncel.2024.1435619

**Published:** 2024-08-30

**Authors:** Keyuan Ren, Qinglong Wang, Douglas Jiang, Ethan Liu, Julie Alsmaan, Rui Jiang, Seward B. Rutkove, Feng Tian

**Affiliations:** ^1^Department of Neurology, Beth Israel Deaconess Medical Center, Harvard Medical School, Boston, MA, United States; ^2^Scripps Institution of Oceanography, San Diego, CA, United States; ^3^School of Arts and Science, Harvard College, Cambridge, MA, United States

**Keywords:** amyotrophic lateral sclerosis, electrophysiology, excitability, excitotoxicity, organoid, disease modeling, electrical impedance myography

## Abstract

Amyotrophic lateral sclerosis (ALS), a devastating neurodegenerative disease, is characterized by progressive motor neuron degeneration, leading to widespread weakness and respiratory failure. While a variety of mechanisms have been proposed as causes of this disease, a full understanding remains elusive. Electrophysiological alterations, including increased motor axon excitability, likely play an important role in disease progression. There remains a critical need for non-animal disease models that can integrate electrophysiological tools to better understand underlying mechanisms, track disease progression, and evaluate potential therapeutic interventions. This review explores the integration of electrophysiological technologies with ALS disease models. It covers cellular and clinical electrophysiological tools and their applications in ALS research. Additionally, we examine conventional animal models and highlight advancements in humanized models and 3D organoid technologies. By bridging the gap between these models, we aim to enhance our understanding of ALS pathogenesis and facilitate the development of new therapeutic strategies.

## 1 Introduction

Amyotrophic lateral sclerosis (ALS) is a devastating neurodegenerative disease characterized by the degeneration of both upper motor neurons (UMNs) in the motor cortex and lower motor neurons (LMNs) in the brainstem and spinal cord. This progressive neuronal loss results in widespread muscle weakness and atrophy, ultimately leading to death due to respiratory failure. ALS has a global incidence of approximately 2 cases per 100,000 person-years (Hardiman et al., 2017). Approximately 5–10% of ALS cases are familial, inherited typically in an autosomal dominant manner, while the majority of cases are sporadic, arising without a clear familial history (Kim et al., 2020; Akcimen et al., 2023). Several factors have been implicated in increasing the likelihood of disease, including sex (men have a moderately higher risk), exposure to certain environmental factors, and military service. However, the disease often appears unexpectedly in otherwise healthy middle-aged individuals (Kiernan et al., 2011; Grad et al., 2017). The progression rate of ALS varies among patients, but most succumb to respiratory failure within approximately 2–5 years of symptom onset. Current therapies of ALS remain very limited, and none of them can fundamentally halt motor neuron loss.

The pathophysiology of ALS remains complex and likely multifactorial. Researchers have observed extensive structural and functional damage to motor neurons, including the degeneration of axons and dendrites. In nearly 97% of patients, ALS is associated with TAR DNA-binding protein 43 (TDP-43) pathology. Affected motor neurons exhibit a shift in TDP-43 protein from cell nuclei to the cytoplasm, where they form dense, skein-like aggregates (Wijesekera and Nigel Leigh, 2009; Grad et al., 2017). However, there is considerable heterogeneity in the pathology of the disease. Mutations in the SOD1 (superoxide dismutase 1) and FUS (fused in sarcoma) genes also cause ALS with very dissimilar pathology (Saccon et al., 2013). Additionally, the most common genetic subtype of ALS is caused by the expansion of a hexanucleotide repeat (GGGGCC) within the C9orf72 (chromosome 9 open reading frame 72) gene, which has also been associated with TDP-43 abnormalities (DeJesus-Hernandez et al., 2011; Renton et al., 2011). This diversity suggests that ALS pathogenesis likely involves multiple pathways, including abnormal protein aggregation within cells and the integrated stress response (Costa-Mattioli and Walter, 2020). These findings underscore the complexity of ALS pathology and may provide valuable directions for potential therapeutic approaches.

One broader pathophysiological concept that has been of interest for some time is excitotoxicity. Excitotoxicity has been proposed as a key underlying mechanism in ALS pathogenesis. For example, spinal motor neurons in mSOD1 mouse embryos demonstrate hyperexcitability (Gunes et al., 2020), which precedes subsequent hypoexcitability and neurodegeneration. This fluctuating excitability pattern is observed in both UMNs and LMNs, highlighting the intricate dynamics throughout the course of the disease. Additionally, there is ongoing debate regarding cortical neuronal hyperexcitability in ALS. It remains unclear whether this hyperexcitability contributes to excitotoxicity or functions as some form of neuroprotective mechanism. Simultaneously, peripheral axonal hyperexcitability has also been described (Bostock et al., 1995), suggesting that this may be a generalized phenomenon contributing to ALS pathogenesis. Denervation, particularly characterized by neuromuscular junction (NMJ) deficits, is another critical pathological mechanism in ALS (Tallon et al., 2022; Verma et al., 2022).

A complex network of pathological changes shapes the progression of ALS. Within the cortical circuitry, there is heightened interconnectivity among motor cortex regions and a concurrent reduction in cortical inhibitory function (Van den Bos et al., 2018). Simultaneously, spinal circuit pathology is characterized by dysregulated neuronal activity mediated by neurotransmitters such as glutamate and GABA/glycine (Ramírez-Jarquín et al., 2014; Brownstone and Lancelin, 2018; Ramírez-Jarquín and Tapia, 2018; Falgairolle and O'Donovan, 2020). Despite extensive research, the specific input circuits primarily contributing to ALS pathology remain uncertain, presenting a crucial area for further investigation. Recent studies have identified glial cells as significant contributors to the disease process, challenging the conventional belief in the intrinsic susceptibility of motoneurons in ALS. The recognition of glial cells as significant contributors to ALS pathogenesis suggests new avenues for therapeutic interventions and further complicates our understanding of ALS (Lasiene and Yamanaka, 2011; Ban et al., 2019; Henstridge et al., 2019).

To investigate the complex pathophysiology of ALS, researchers have developed various experimental models, including both animal and humanized models. Animal models, such as transgenic mice expressing mutant forms of human genes (e.g., SOD1, TDP-43, FUS, and C9orf72), have been instrumental in studying disease mechanisms and testing potential therapies (Philips and Rothstein, 2015). These models mimic many aspects of ALS pathology, including motor neuron degeneration, protein aggregation, and neuroinflammation. However, they also have limitations, such as differences in disease progression and pathology compared to human ALS, prompting a shift towards humanized models like human-induced pluripotent stem cells (iPSCs), which can differentiate into motor neurons and other relevant cell types, thereby providing a more accurate system to study ALS (Bhargava et al., 2022). The advent of iPSC technology enables investigation of patient-specific genetic background and their impact on ALS pathology, thereby offering broad applications in disease modeling, cell therapy, and drug screening and aiding in the identification of patient-specific therapeutic targets (Fujimori et al., 2018; Du et al., 2023). More recently, 3D organoid models have emerged as a promising tool for ALS research. These models offer a more physiologically relevant environment compared to conventional 2D cultures, allowing the study of complex cell–cell interactions and tissue architecture (Sun X. et al., 2018). Organoids derived from ALS patient iPSCs can recapitulate key features of the disease, such as TDP-43 aggregation and neuronal hyperexcitability, providing valuable insights into ALS mechanisms and potential therapeutic targets (Tamaki et al., 2023).

Another important challenge in ALS research is effectively tracking disease deterioration in both the laboratory and clinical settings so as to understand progression rates and the effects of potential therapies (Chipika et al., 2019). A variety of approaches and biomarkers have been suggested over the years. Clinically, these include imaging studies, such as magnetic resonance imaging (MRI) to evaluate brain, spinal cord, or muscle volume, and ultrasound to measure muscle thickness (Grolez et al., 2018; Leoni et al., 2022). Serological assays such as assessment of serum neurofilament heavy, neurofilament light, and cystatin C, have also been advocated (Steffen et al., 2022; Zhu et al., 2023b). In the laboratory, such tools are less critical since cells can be directly visualized and characterized, and animals can be euthanized and disease status easily quantified. Still, animal biomarkers that are relatively translatable to humans can be helpful in a variety of ways, including therapy validation. Among these tools, electrophysiological techniques are especially useful due to their flexibility and relevance. Indeed, given the profound alterations in motor neuron function and number and the consequent alterations in the NMJ and myofiber, electrophysiologic approaches have remained a foundational approach for assessing basic pathophysiology, disease progression and potential response to new therapies.

In this review, we explore the range of electrophysiological tools available for studying ALS. We discuss the integration of these tools with various ALS models, including conventional animal models and emerging humanized and 3D organoid models, highlighting their important role in tracking disease progression and investigating underlying mechanisms.

## 2 The electrophysiological tools to synergize disease modeling of ALS

Electrophysiological assessments can be generally categorized into cellular and organismal techniques. Cellular electrophysiology includes tools such as patch clamp, calcium imaging, and multielectrode arrays (MEAs) (Neher and Sakmann, 1992; Grienberger and Konnerth, 2012; Soscia et al., 2020). These tools have been used to elucidate many primary features of ALS, including abnormalities in single motor neuron resting membrane potential and specific channel dysfunctions (Wainger et al., 2014). Tools for organismal assessment can be applied to humans as well as animal models and include entities such as motor unit number estimation (MUNE), excitability testing, and electrical impedance myography (EIM) (Shefner et al., 2011; Sanchez and Rutkove, 2017; Wainger et al., 2021). Organoid technology represents a unique intersection in this classification. While organoids can be studied using cellular electrophysiological techniques to explore cellular responses and interactions, they also bridge the gap to organismal-level studies. This is due to their ability to mimic tissue and organ structures, allowing for complex tissue-level electrophysiological investigations (Qian et al., 2019).

### 2.1 Cellular electrophysiological tools

#### 2.1.1 Patch clamp

**Figure 1:**
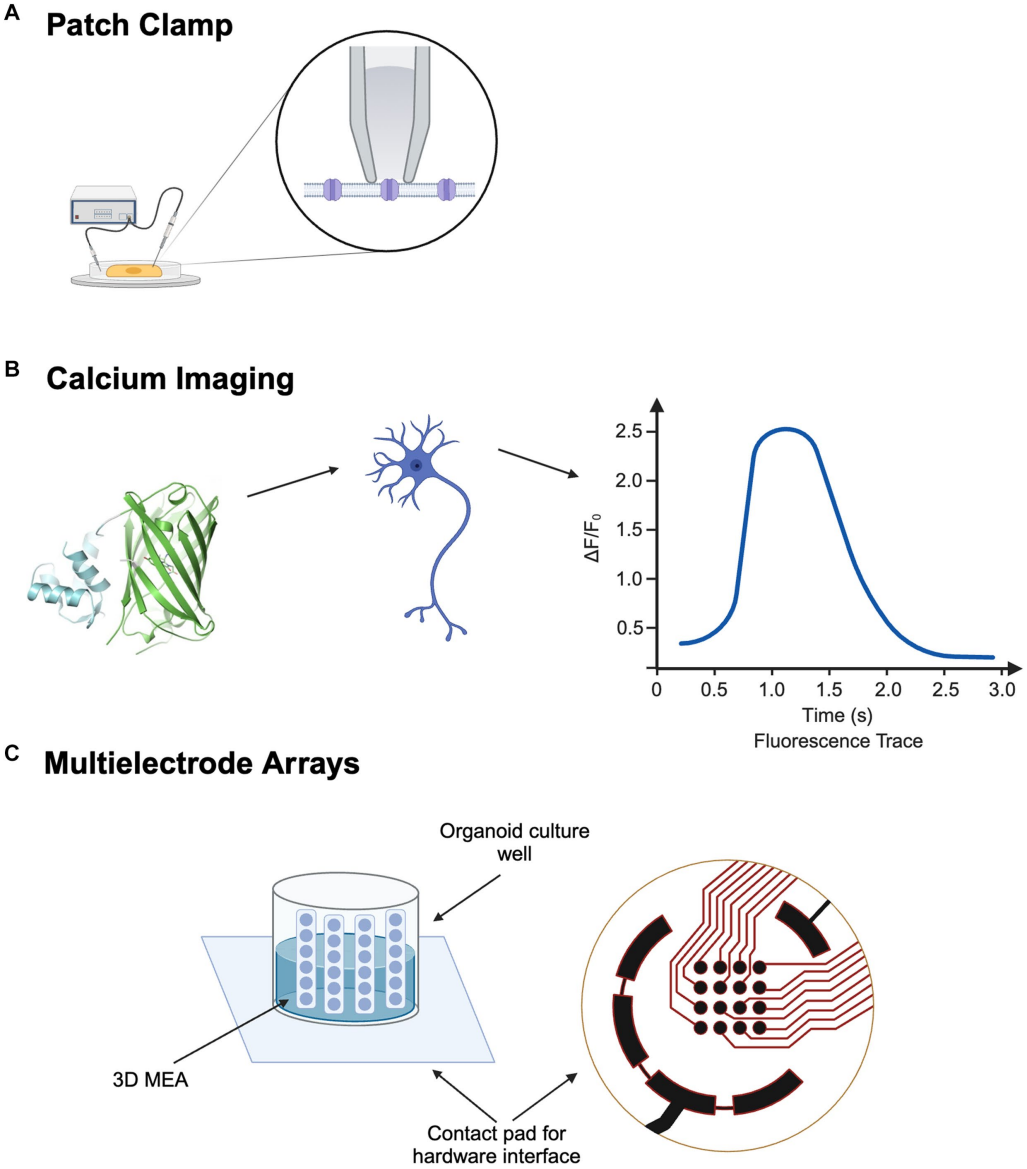
Electrophysiological Approaches for Cellular and Network Analysis in ALS: **(A)** Patch Clamp for ion channel profiling. **(B)** Calcium Imaging for neuronal network activity. (C) 3D Multi-electrode Array. Created with BioRender.com.

Ion channels, embedded in the cell membrane, are responsible for signal transmission between and within cells (Neher and Sakmann, 1992). These transmissions are typically fostered by the movement of cations such as Na^+^, K^+^, and Ca^2+^ (Kornreich, 2007). The patch clamp technique involves creating a high-resistance seal on a patch of the cell membrane with a glass micropipette, allowing for the precise measurement of electrical activity of a single cell, even across a single ion channel (Figure 1A). This technique provides exceptional temporal resolution, making it ideal for capturing rapid changes in ion channel activity (Passaro and Stice, 2020). For example, Yang et al. used whole-cell patch clamp on LMNs derived from iPSCs from day 18 to 28 of differentiation and measured significantly more action potentials on day 28 (Yang et al., 2023). Wainger et al. also used patch clamp on iPSC-derived motor neurons on day 28, observing a significant increase of minimal current step that triggers action potential with retigabine, which reduced hyperexcitability (Wainger et al., 2014). Despite its many benefits, achieving low-noise measurements with the patch clamp can be extremely laborious, requiring delicate manipulation of the pipette and detailed fabrication of the electrodes (Zhao et al., 2008). While providing exceptional temporal resolution, patch clamp offers little spatial resolution, making it less suitable for studying large networks of neurons (Passaro and Stice, 2020).

#### 2.1.2 Calcium imaging

Calcium ions play an essential role in a myriad of cellular activities across virtually all cell types (Berridge et al., 2000). By leveraging numerous fluorescent calcium indicators, calcium imaging allows the visualization and measurement of the status of calcium cations in cells (Figure 1B) (Grienberger and Konnerth, 2012). This technique is particularly instrumental in mapping neural activities, as neuronal firing is closely linked to fluctuations in intracellular Ca^2+^ levels. While calcium imaging offers compromised temporal resolution, it compensates with higher spatial resolution, providing large-scale activity information (Passaro and Stice, 2020). When integrated with optogenetics, calcium imaging significantly enhances the analysis of neural circuits within organoids, mapping and elucidating interactions among different pathways by manipulating ion channels (Stamatakis et al., 2018). This combined methodology has shown potential in drug discovery and screening by enabling precise observation of drug effects on neural activity and calcium dynamics. However, despite these advantages, calcium imaging’s less optimal temporal resolution can be a limitation for capturing rapid changes in cellular activity (Peterka et al., 2011).

#### 2.1.3 MEAs for detection of neuronal and myofiber action potentials

Neurons can be plated onto MEAs, where microelectrodes are embedded on the bottom of the dish (Figure 1C). After axons and dendrites form uncountable synaptic connections, the MEA acts as a neural interface where network activity can be manipulated by artificial electrical stimulations (Hales et al., 2010). While effective for 2D cultures, conventional MEAs face limitations with 3D organoids (Passaro and Stice, 2020). To combat this limitation, 3D MEAs have been developed. With the addition of implantable MEA probes, vertical cross-sections of the 3D organoid can be measured with increasing spatial and temporal precision (Lam et al., 2021). For example, Soscia et al. developed a polyimide-based 3D MEA, where the vertical positions of the probes were held by the mechanical forces of plastic deformation. Recordings were conducted over 38 days on a human iPSC neuronal culture. Electrodes began to pick up electrophysiological signals by day 15, and 70 out of 80 electrodes across three arrays were active at the end of 38 days (Soscia et al., 2020). While the insertion of probes allows MEA to measure electrical activity across 3D cultures, it may also inflict considerable harm to the neural network. Recent advancements in flexible bioelectronics have led to the creation of soft, conformable electronic devices, where a 2D electronic device can be folded into a 3D structure, allowing it to delicately wrap around 3D brain organoids and facilitate a less invasive electrical interface (Tasnim and Liu, 2022). Wainger et al. used a 64-electrode MEA to compare action potentials in iPSC-derived motor neurons from ALS and control subjects, revealing more spontaneous action potentials in ALS samples (Wainger et al., 2014).

### 2.2 Clinical electrophysiological tools

On the clinical side, both hyperexcitability and hypoexcitability have been described as features of ALS, with corresponding tools, including peripheral nerve excitability testing and transcranial magnetic stimulation (TMS) being extensively studied (Suzuki et al., 2022; Verma et al., 2022; De Carvalho and Swash, 2023). The assessment of motor neuron number has been pursued for even longer, utilizing methods such as motor unit number estimation (MUNE) and motor unit number index (MUNIX) (Shefner et al., 2011; Jacobsen et al., 2018). NMJ abnormalities can be quantified via repetitive nerve stimulation or jitter analysis via single fiber electromyography (Sanders et al., 2022; Zhu et al., 2023a). And finally, the consequent atrophied state of skeletal muscle can be quantified using EIM (Sanchez and Rutkove, 2017).

#### 2.2.1 MUNE/MUNIX

**Figure 2:**
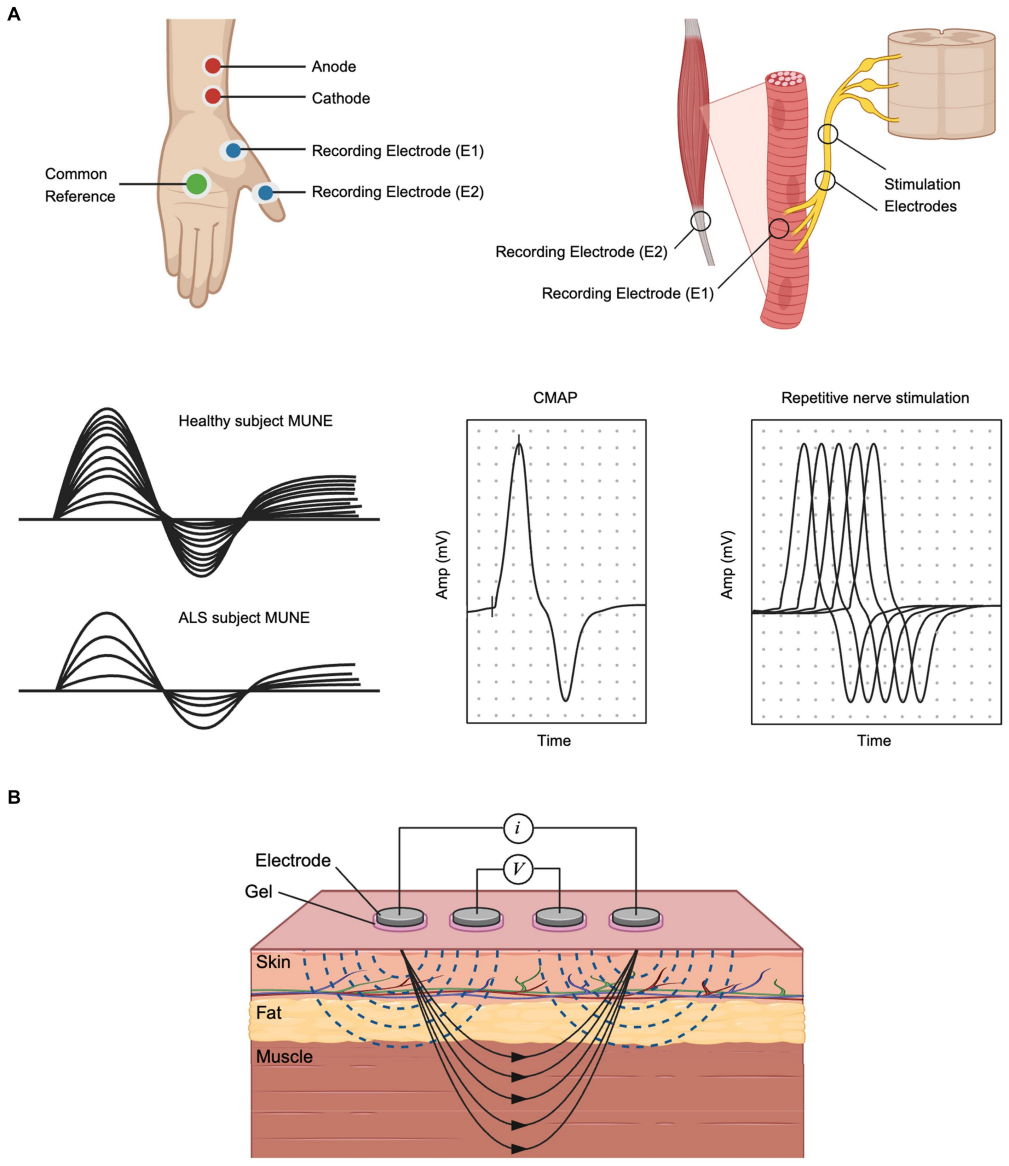
Electrophysiological Approaches for Structural and Functional Analysis in ALS: **(A)** MUNE and CMAP recording setup and typical MUNE, CMAP and RNS waveforms. Stimulation electrodes are placed on the nerve, while recording electrodes are placed on the muscle. **(B)** Electrical Impedance Myography. Created with BioRender.com.

Reliable outcome measurement tools are needed to accurately diagnose ALS and assess therapy effectiveness. Assessment tools such as the ALS Functional Rating Scale-Revised (ALSFRS-R) or handheld dynamometry have been used in many clinical trials but are limited by variability between research subjects and reliance on patient motivation and mood (Benatar et al., 2016). MUNE and MUNIX offer interesting alternatives, since they provide quantitative assessments of collective motor unit number and health that are entirely independent of patient mood or motivation (Figure 2A) (Gawel, 2019). A study comparing ALSFRS-R to MUNE demonstrated a greater mean rate of decline per year as well as a lower coefficient of variation for MUNE, supporting its value over ALSFRS-R in future clinical trials (Shefner et al., 2011). The individual motor unit potential (MUP) amplitude or area is another measure that shows changes with reinnervation in ALS. However, evaluating the MUP does not provide information about motor unit loss and only MUPs produced by early recruited motor units can be measured (Jacobsen et al., 2018). MUNIX, offers a simpler, more automated approach for obtaining an index (versus an estimation) of the number of functioning motor units and continues to be studied as a useful alternative approach (Fatehi et al., 2018). Finally, the compound motor action potential (CMAP) itself is sensitive to decline and can be followed over time (Figure 2A) (Shefner et al., 2011).

#### 2.2.2 Excitability testing

Both central and peripheral hyperexcitability and hypoexcitability are characteristic features of ALS; therefore, tools such as TMS and neuronal excitability testing (NET) are valuable for understanding ALS pathogenesis, progression, and therapy impact. TMS techniques such as cortical motor threshold (CMT) and the motor evoked potential amplitude (MEP) have been used to study disease progression in ALS (Wang et al., 2017; Yu et al., 2021). However, the sample size of these TMS studies is small and lack inter-test comparisons and multi-center reproducibility (De Carvalho and Swash, 2023). TMS measurements are also limited by progressive muscle degeneration and increasing cortical inexcitiability as ALS progresses. Therefore, TMS studies, such as peristimulus time histogram (PSTH), cortical silent period (CSP), and short-interval intracortical inhibition (SICI), which investigate cortical inhibition evaluate early disease-course abnormalities, show promise (Wainger et al., 2021; De Carvalho and Swash, 2023). Studies using TMS and median nerve excitability testing have revealed that patients with ALS exhibit both cortical and peripheral hyperexcitability compared to controls; additionally, only peripheral hyperexcitability and not cortical hyperexcitability is associated with the extent of peripheral burden (Suzuki et al., 2022).

#### 2.2.3 NMJ testing: repetitive stimulation and single fiber electromyography

Repetitive nerve stimulation (RNS) is a commonly applied assessment of NMJs (Figure 2A). It has been known since 1959 that decremental responses in low-frequency RNS (LF-RNS) are a common phenotype in ALS patients (Sun X. S. et al., 2018). Studies applying LF-RNS to study regions and patterns of decremental response have found that degeneration of NMJ could occur before that of motor neurons, and damage of the NMJ occurred at both post-and pre-synaptic membranes (Sun X. S. et al., 2018). Similarly, single fiber electromyography has long been known to be an early finding of denervation with resulting NMJ instability (described as increased jitter) (Liu et al., 2013). However, given the time intensive nature of this procedure, it is not typically used in the clinical setting to diagnosis ALS or evaluate its progression.

#### 2.2.4 Electrical impedance myography (EIM)

EIM is a non-invasive bioimpedance-based technique that evaluates neuromuscular health by recording voltages from muscle tissue in response to a weak, high-frequency electrical current (Figure 2B) (Sanchez and Rutkove, 2017). Changes in the composition and histology of muscle, which are common to nearly all neuromuscular disorders, including ALS, impact the impedance properties of the tissue, which can be readily captured by technology. Indeed, EIM is especially sensitive to denervation-associated atrophy of ALS, making it a biomarker uniquely sensitive to disease progression in ALS (Shefner et al., 2018). Critically, unlike nearly all standard electrophysiologic measures, EIM does not measure the innate electrical properties of the muscle (e.g., myofiber depolarization). Accordingly, EIM can be readily performed on *ex vivo* muscle tissue, which has been a major area of study, showing alterations in muscular dystrophy animal models (Li et al., 2014; Pandeya et al., 2021), disuse/microgravity analogues (Semple et al., 2020), and sciatic crush (Ahad et al., 2009). It can also even be performed on very small animals, such as zebrafish (Rutkove et al., 2023b). Given the simplicity of measurement, EIM can be readily adapted to be used on human organoids that include myofibers as a component, providing a rapid assessment of the integrity and health of the tissue. Indeed, the same MEAs that are used to perform standard electrophysiological analysis can also be used for EIM, with 2 (or more) electrodes being selected for current application and a separate 2 (or more) electrodes selected for voltage measurement. Finally, we note that while in the context of ALS, impedance techniques have been used predominantly on muscle, there is nothing precluding their use on brain organoids as well.

## 3 Conventional animal models of ALS

With the identification of multiple ALS susceptibility genes, various gene-edited animal models have been employed for investigating ALS pathology. While these animal models offer certain research advantages, they also possess inherent limitations (Table 1). Several animal models, from the most primitive to most complex, commonly used in ALS research and the potential applications of neurophysiology are described here.

**Table 1:** Comparative overview of animal and humanized models in ALS research.

Model type	Genetic consistency with humans	Life cycle and manipulability	Pathological features modeled	Electrophysiological techniques applied to the model	Advantages	Limitations and challenges	Reference
Roundworm (*Caenorhabditis elegans*)	Highly conserved	Short, easy to manipulate	Axonal degeneration, NMJ pathology	Patch clamp, calcium imaging, NMJ testing	Cost-effective, simple genetics, rapid life cycle	No visceral metabolic pathways, no myelin sheath	Goodman et al. (2012)
Fruit fly (*Drosophila melanogaster*)	Partially conserved	Short, easy to manipulate	Neuron degeneration, cellular anomalies	Patch clamp, calcium imaging, MEAs, NMJ testing	Well-established genetic tools, short life span	Different brain and organ anatomy, no adaptive immune system	Bykhovskaia and Vasin (2017) and Liguori et al. (2021)
Zebrafish (*Danio rerio*)	Highly conserved	Short, easy to manipulate	ALS progression during development	Patch clamp, calcium imaging, MEAs, NMJ testing, EIM	Transparent embryos, easy to manipulate, rapid development	No clear connection between cortical and spinal motor neurons	Chia et al. (2022)
Mouse (*Mus musculus*)	Highly conserved	Longer, more complex	Neuroanatomical, cardiovascular similarities	Patch clamp, calcium imaging, MEAs, MUNE, NMJ testing, EIM	Well-established model, genetic manipulation capabilities, similar neuroanatomy	Genetic differences, limited replication of human disease pathology, treatment efficacy issues in human trials	Lutz (2018), Rodriguez et al. (2021), and Van Schoor et al. (2022)
Rat (*Rattus norvegicus*)	Highly conserved	Longer, more complex	Similar to mouse, larger tissue availability	Patch clamp, calcium imaging, MEAs, MUNE, NMJ testing, EIM	Larger brain size for easier surgical manipulation, similar neuroanatomy	Similar to mouse	Howland et al. (2002) and Lagier-Tourenne and Cleveland (2009)
Non-human Primates	Highly conserved	Long, complex, ethical considerations	Genetic, physiological similarities	Patch clamp, calcium imaging, MEAs, NMJ testing	Closest physiological and genetic resemblance to humans, comprehensive behavioral studies	Ethical considerations	Bang et al. (2022) and Xu L. et al. (2023)
iPSC Models	Human-derived	Variable, lab-dependent	Hyperexcitability and hypoexcitability phenotype	Patch clamp, calcium imaging, MEAs, NMJ testing	Patient-specific, can model genetic diversity, scalable for high-throughput screening	2D limitations, apoptosis alterations	Burley et al. (2022) and Ronchi et al. (2021)
3D Organoids	Human-derived	Variable, lab-dependent	Brain immune-related signaling	Patch clamp, calcium imaging, MEAs	Mimics *in vivo* tissue architecture, can study complex cell interactions, long-term culture potential	Lack of vascular cells, reliance on diffusion	Burley et al. (2022), Eremeev et al. (2021), and Keller and Frega (2019)

### 3.1 Caenorhabditis elegans

The complete anatomical structure of the *C. elegans* cell lineage and gene sequence has been deciphered, revealing a highly conserved gene sequence with humans. As a disease model, *C. elegans* possesses characteristics such as a short life cycle, simple gene editing capabilities, and a transparent whole life cycle, making it an ideal simplified model for long-term observation research on chronic diseases. A variety of ALS models have been established in *C. elegans*, including C9orf72, SOD1, TDP-43, and FUS models (Roussos et al., 2023). Based on the *C. elegans* model, Yoshifumi Sonobe and his colleagues demonstrated a conserved role for eif-2D/elF2D in dipeptide repeats expression (Sonobe et al., 2021). Additionally, axonal degeneration and NMJ pathology are also reproduced in the *C. elegans* model (Markert et al., 2020; Baskoylu et al., 2022; Tossing et al., 2022). Despite its advantages, the nematode model has significant limitations, such as the absence of visceral metabolic pathways, a blood–brain barrier, and myelinated neurons.

A variety of electrophysiological tools have been employed to unravel the complexities of neural and muscular functions in *C. elegans*. Patch-clamp techniques have been pivotal in studying ion channel functions and electrophysiological properties of neurons and muscle cells, providing insights into the modulation of neuronal function and mechanotransduction (Ronan and Gillespie, 2005). Calcium imaging is another critical tool, used to visualize intracellular calcium dynamics and understand neuronal signaling and muscle contractions, illuminating the role of calcium channels in these processes (Goodman et al., 2012). Other electrophysiological approaches, such as MEA, EIM, or MUNE have yet to be attempted given the organism’s very small size.

### 3.2 Drosophila melanogaster

*Drosophila*, commonly known as the fruit fly, emerges as a pivotal genetic model, offering profound insights into disease mechanisms. The utility of *Drosophila* is anchored in its amenability to straightforward genetic manipulation, diverse methodologies for drug administration, which includes feeding, injection, as well as various assays that could be used to evaluate disease progression, including climbing and crawling tests, eclosion rate, phenotypical and electrophysiological observations and screenings. These methodologies facilitate a comprehensive understanding of ALS pathogenesis, closely mirroring human symptomatology in aspects of survival, motor function, neuron degeneration, and cellular and mitochondrial anomalies. A unique feature of *Drosophila* in ALS studies are its visual system. Approximately two-thirds of vital genes in the *Drosophila* genome play roles in eye development, rendering the fly’s eye a potent tool for studying neurodegeneration. The eye’s structured array of approximately 800 ommatidia, each comprising photoreceptor neurons and pigment cells, allows for the discernment of subtle genetic alterations manifesting as morphological aberrations, thus enabling the investigation of potential genetic modifiers, including C9orf72, SOD1, FUS, TDP-43 and Ataxin-2 (Casci and Pandey, 2015).

However, limitations in this model must be acknowledged. The fruit fly’s brain and major organ anatomy markedly differ from humans, and it lacks an adaptive immune system. Furthermore, the absence of complex cognitive abilities in flies can constrain behavioral studies. Additionally, divergent drug responses between *Drosophila* and humans necessitate comparative analyses across various animal models. Despite these constraints, *Drosophila* remains an invaluable organism for rapid, cost-effective genetic screening, and elucidating ALS’s genetic and mechanistic pathways, thus bolstering the search for novel diagnostics and therapeutics for ALS (Liguori et al., 2021).

Patch-clamp techniques have been refined for *Drosophila* to allow recordings from specific neuronal types, enabling a detailed study of the electrical properties of fly neurons. For example, Fernández-Chiappe and Muraro provided a comprehensive protocol for mastering this technique in *Drosophila* brain neurons, highlighting its utility in neuroscientific research (Fernandez-Chiappe and Muraro, 2022). Calcium imaging is another critical tool in *Drosophila* research, used to observe intracellular calcium dynamics. Maimon et al. adapted this technique for tethered flying and walking *Drosophila*, revealing how the gain of visual motion–processing interneurons changes with locomotor state (Maimon et al., 2010). As with *C. elegans,* the small size of *Drosophila* makes it challenging to study with techniques typically geared for larger animals.

### 3.3 *Danio rerio* (zebrafish)

As vertebrates, zebrafish are phylogenetically closer to humans than nematodes or *Drosophila*, and can be easily subjected to simple behavioral tests (Oliveira et al., 2023). As an emerging ALS model organism, zebrafish offer advantages such as relatively easy genetic regulation, highly conserved disease-related genes, and efficient drug screening compared to other vertebrate models. Additionally, the transparency of zebrafish embryos provides a significant advantage for observing ALS progression during development (Morrice et al., 2018). The zebrafish genome and connectomes have been completely mapped, making them an ideal simplified model for studying signaling pathways (Howe et al., 2013; Kunst et al., 2019; Zhang et al., 2021). Currently, commonly used zebrafish ALS models include SOD1, TDP-43, FUS, C9orf72, and other environmental neurotoxins exposure models (Chia et al., 2022). ALS models in zebrafish can be achieved through chemical induced pathological alterations (Powers et al., 2017; Roy et al., 2017; Martin et al., 2021) or through the expression of disease-related gene mutations in systematic pathways such as the integrated stress pathway (Fortier et al., 2020; Campanari et al., 2021; Petel Legare et al., 2023). These models have revealed mechanisms underlying multiple signaling pathways (Shaw et al., 2018; Swinnen et al., 2018; Pant and Nazarko, 2021). However, zebrafish lack a clear connection between cortical and spinal motor neurons; thus, while very effective at mirroring lower motor neuron aspects of the disease, their relevance to upper motor neuron pathology is far less certain.

Patch-clamp recordings have been effectively used in zebrafish to study spinal neurons and their development. For example, Saint-Amant and Drapeau described a preparation for obtaining patch-clamp recordings from embryonic spinal cord interneurons, motoneurons, and sensory neurons in zebrafish, allowing the study of spontaneous and touch-evoked electrical activity during spinal cord development (Saint-Amant and Drapeau, 2003). Calcium imaging is another significant tool; Brette et al. presented a method for isolating ventricular myocytes from zebrafish heart and used calcium imaging to study their electrophysiological characteristics, which is pertinent for cardiac alteration research in ALS (Brette et al., 2008). EIM has now been successfully applied to zebrafish to evaluate the effects of aging (Rutkove et al., 2023a,b), and studies of ALS progression mutant SOD1 zebrafish are currently ongoing (Rutkove et al., unpublished results). Other standard clinical neurophysiological studies may have potential to be applied to zebrafish, such as obtaining motor unit potentials, MUNE, and single fiber electromyography (EMG).

### 3.4 *Mus musculus* (mouse)

The mouse remains the most commonly utilized animal model for studying ALS due to its neuroanatomical and cardiovascular similarities with humans, making it extensively employed in ALS pathological investigations, drug screening, and clinical trials (Morrice et al., 2018). The optimization of gene editing technology and the emergence of tools such as circuit-specific virus labeling have enabled more efficient investigation into the contribution of specific signaling pathways to ALS disease progression in mouse models (Lee et al., 2020; Rodriguez et al., 2021; Van Schoor et al., 2022; Ziff et al., 2022). With the intact brain-spine-muscle axis preserved in the mouse model, further investigations can systematically elucidate the underlying mechanisms involved in the pathological development of ALS (Quarta et al., 2018; Haney et al., 2019; Bayer et al., 2021; Amalyan et al., 2022). Although studies in mice have provided insights into various mechanisms underlying ALS, treatments developed based on mouse models often fail to demonstrate efficacy in human disease (Bonifacino et al., 2021). Part of the reason for this is that most of these models rely on over expression of toxic molecular components, which may not accurately mimic human disease.

The patch-clamp technique is a cornerstone in this research, with automated systems developed for *in vivo* applications in the mouse cortex and hippocampus (Kodandaramaiah et al., 2012). Calcium imaging is also instrumental; for example, Ladewig and Keller used it to study calcium oscillations in motoneurons, which is important for understanding ALS (Ladewig and Keller, 2000). Given this animal’s larger size than those listed earlier, MUNE (Shefner et al., 2006) and EIM (Li et al., 2013) have both been applied.

### 3.5 Other animal models

Including rats, dogs, and non-human primates, have significantly contributed to our comprehension of ALS pathophysiology and the evaluation of potential therapeutic avenues. Rats, with their larger body size and greater tissue availability, have served as valuable models in ALS research. A recent study showcased a robust correlation between EIM and MUNE data with ALS progression in rat models (Wang et al., 2011). Notably, both EIM and MUNE exhibited strong associations with survival, with EIM phase-slope emerging as the most reliable indicator of disease progression. Degenerative myelopathy (DM) is a late adult-onset, progressive neurodegenerative condition in dogs that shares similarities with some forms of SOD1-associated human ALS. Studies on DM in dogs explore the utility of EIM in assessing muscle pathology, demonstrating that EIM, particularly 100 kHz phase values, offers sensitive detection of muscle pathology in DM-affected dogs. This highlights the potential of EIM as a valuable tool for monitoring disease progression and evaluating therapeutic interventions in veterinary medicine (Kowal et al., 2022). Another study examines the feasibility of MUNE in canine neurology, measuring the number of motor units in healthy dogs and those with various neurological disorders (Kauder et al., 2015). Non-human primates have also been utilized as animal models in studying ALS, as primates share many genetic, developmental, and physiological similarities with humans (Bang et al., 2022). Researchers using Cynomolgus monkeys have been able to create a primate model of C9orf72 related dipeptide repeats (DPRs) and show that poly (PR) DPRs are sufficient to induce C9-ALS/frontotemporal dementia (FTD)-like symptoms in primates (Xu L. et al., 2023). Additionally, researchers using Cynomolgus monkeys with overexpressed human wild-type TDP-43 in their spinal motoneurons have been able to detect TDP-43 mislocalization in almost all the large motoneurons of the lateral nuclear group at the early or even the presymptomatic stage. This finding is consistent with the observation that, in human ALS patients, the highest percentage of neurons with TDP-43 mislocalization is found in the early stages of the disease. In contrast, mouse models seem to show that overexpression of wild-type TDP-43 in the nuclei is sufficient to be toxic to spinal motoneurons (Uchida et al., 2012). These inconsistent results across different animal models may indicate that TDP-43 mediated pathology is variable across animal species and that human-relevant pathology may be more accurately modeled in species more closely related to humans. However, it is important to note the limited published data on using standard electrophysiological tools in non-human primates. A recent thesis has detailed a novel approach using rhesus macaques, where the overexpression of human TDP-43 in selective spinal motoneuron populations induced motor neuron disease (MND)-like pathogenesis (Jones, 2022). The study used clinical monitoring techniques such as MRI, EMG, and nerve stimulation, revealing a progression of changes analogous to those observed in human MND. While these findings provide compelling evidence of shared MND-like pathogenesis in primates, further research is needed to comprehensively evaluate the utility of these electrophysiological tools in non-human primate models.

## 4 Humanized models of ALS and translational applications of 3D organoids

In short, the expression of ALS mutations in animal models often fails to accurately replicate the diverse phenotypes observed in human disease. Given the limitations of animal models in disease treatment, there has been a growing emphasis on utilizing humanized models for research purposes (Bhargava et al., 2022). Differences in epigenetics between animal models and the human brain, as well as variations in cell types and neural networks, contribute to the limited predictive ability of animal models for drug efficacy. Additionally, the human brain exhibits longer axons and a greater number of synaptic connections, which pose challenges when attempting to replicate these features in animal models (Okano and Morimoto, 2022).

### 4.1 iPSC and patient derived-iPSC for disease modeling

iPSCs are pluripotent stem cells generated by introducing specific transcription factors into somatic cells. This reprogramming process allows the cells to acquire the morphology and growth properties of embryonic stem cells (ESCs) (Takahashi and Yamanaka, 2006). iPSCs are invaluable for disease research due to their capacity for continuous differentiation and self-renewal (Chehelgerdi et al., 2023). Before iPSC-based models were developed, ESCs were the primary pluripotent stem cells used in research. However, ESCs are challenging to obtain and present ethical concerns regarding the use of embryos. iPSCs circumvent these ethical issues and offer a robust, patient-specific platform for disease modeling, drug screening, and cell therapy development (Cerneckis et al., 2024). This is particularly advantageous for studying diseases like ALS, where patient-derived iPSCs can be differentiated into motor neurons, enabling precise disease modeling and therapeutic testing.

iPSC models hold significant potential for advancing pathological research and facilitating drug prediction. Electrophysiological characterization of human iPSC-derived motor neurons with SOD1 mutations and C9orf72 repeat expansions have revealed a membrane hyperexcitability phenotype (Burley et al., 2022). Moreover, research conducted on human iPSC-derived neurons, specifically motor neurons carrying the TDP-43 Q331K mutation associated with ALS, has shown a decrease in the firing rate of action potentials (Ronchi et al., 2021). The current state of research demonstrates the successful generation of phenotypes associated with ALS mutations using iPSC models, leading to the elucidation of multiple signaling pathways and biomarkers in patients (Badu-Mensah et al., 2020; Li et al., 2022; Zhang et al., 2022).

Following the introduction of 3D organoid models, iPSCs have gained some utility in the intersection of organoids and iPSCs. With recent advances with the application of iPSCs in disease research, iPSCs have been incorporated in the generation of 3D organoid models to form iPSC-derived 3D organoid models. These organ-specific iPSC-derived 3D organoid models capture more *in vivo* characteristics of cells than conventional two-dimensional *in vitro* models while removing limitations of imaging difficulty, confounding variables, and availability constraints that animal models often have (Xu Z. et al., 2023).

### 4.2 iPSC-based 2D models

iPSC-based 2D models are highly valued by researchers for their efficiency, cost-effectiveness, and ethical benefits. These models are particularly useful for high-throughput screening and detailed cellular analysis. However, they have notable limitations, including reduced cell–cell interactions and the inability to mimic the complex three-dimensional structure of tissues. This simplification can result in altered cellular behaviors, such as changes in apoptosis pathways (Yamada and Cukierman, 2007). Despite these challenges, iPSC-based 2D models continue to be instrumental in early-stage disease modeling and drug discovery, providing critical insights into cellular responses and disease mechanisms.

### 4.3 3D organoid models

**Figure 3:**
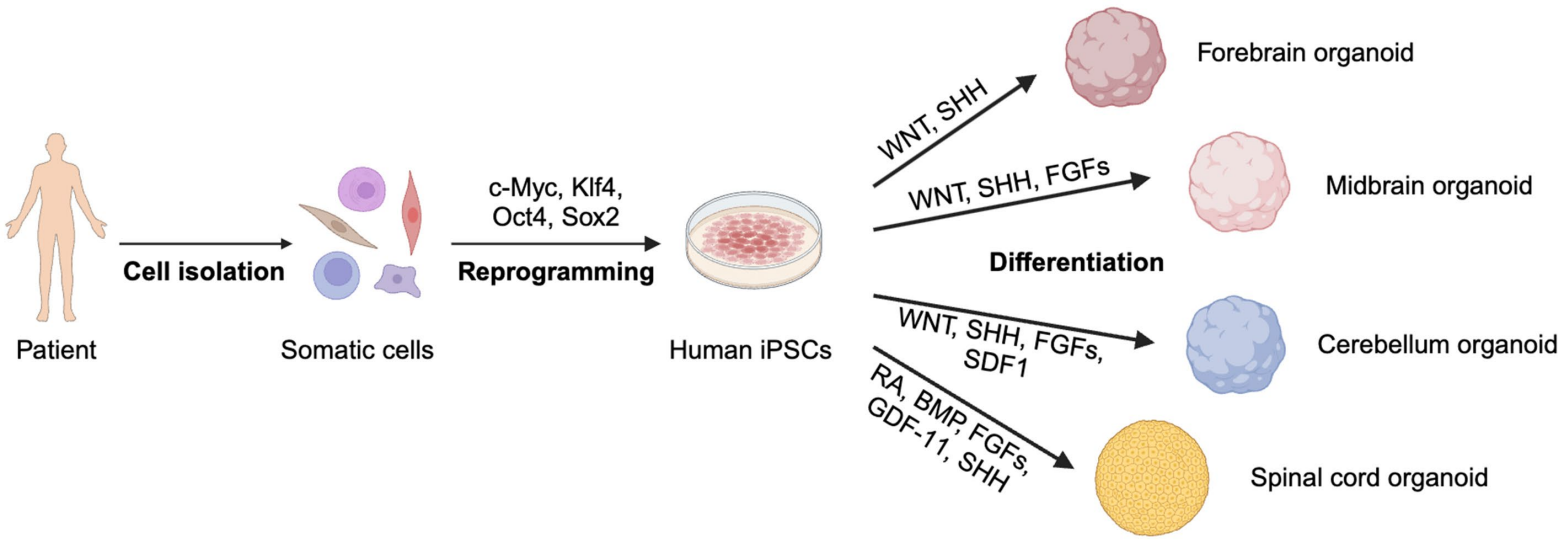
Generation of 3D organoids from patient-derived iPSCs. Somatic cells are reprogrammed into iPSCs, which differentiate into forebrain, midbrain, cerebellum, and spinal cord organoids using specific signaling factors. Created with BioRender.com.

Compared to 2D iPSC models, 3D organoids offer a more faithful representation of the physiological architecture of *in vivo* tissues, exhibiting not only different cell types but also structural characteristics (Figure 3). For instance, 3D organoids can replicate human cortical folds. Leveraging the structural advantage of organoids, an increasing number of studies have been dedicated to investigating neuron–neuron and neuron-astrocyte communication in organoid models (Meijboom et al., 2022; Wang et al., 2022; de Majo et al., 2023).

Recent research has demonstrated the successful generation of long-term cultures of cortical brain organoid slices (cBOS), revealing mature neurons and astrocytes organized in complex architecture. Employing whole-cell patch-clamp and calcium imaging techniques, researchers observed subthreshold synaptic inputs, action potential firing, and synchronous large-scale oscillations in cBOS (Petersilie et al., 2024). This method offers the potential to investigate the hyperexcitability and hypoexcitability characteristics of motor neurons in ALS.

In addition to cBOS, recent advancements in intact 3D organoid models have shown significant promise. Intact 3D organoids maintain their structural integrity without being sectioned into slices, allowing for more comprehensive studies of tissue-level electrophysiological properties. For example, human cortical spheroids, a type of intact 3D organoid, sustain spontaneous excitatory postsynaptic currents (sEPSCs), exhibit action potentials, and display complex network activities, making them valuable for modeling cortical development and neurological diseases (Pasca et al., 2015). These studies have demonstrated that intact organoids can exhibit more complex and physiologically relevant behaviors compared to cultured slices, such as long-range neuronal connectivity and three-dimensional tissue interactions.

Moreover, recent advancements in MEAs have overcome limitations posed by organoid complexity, allowing precise detection of neuronal and myofiber action potentials (Soscia et al., 2020). These innovations enable less invasive interfaces with 3D brain organoids, highlighting differences in spontaneous activity between ALS patients and controls (Tasnim and Liu, 2022). Particularly sensitive to denervation-associated atrophy in ALS, EIM serves as a unique biomarker for disease progression. Its simplicity suggests potential for future implementation in human organoids containing myofibers, enabling rapid assessments of tissue health and integrity. Overall, organoid models hold the potential to uncover how ALS impacts neuronal electrophysiology, aiding in understanding disease progression and developing targeted interventions. These advancements highlight organoids’ transformative role in elucidating disease pathophysiology and advancing novel therapies.

### 4.4 Translational applications of 3D organoid

3D organoids offer significant advantages in translational research due to their ability to replicate the complex architecture and cellular diversity of human tissues (Velasco et al., 2019). These models provide a more physiologically relevant environment compared to conventional 2D cultures, enabling the study of intricate cell–cell interactions, tissue organization, and functional properties (Lancaster and Knoblich, 2014; Quadrato et al., 2017; Levy and Pasca, 2023). 3D organoids derived from patient-specific iPSCs can recapitulate key pathological features of ALS, such as protein mislocalization and aggregation (Ratti et al., 2020). These models facilitate the testing of new therapeutic compounds and enhance the translational relevance of preclinical findings, thus improving drug discovery efforts (Keller and Frega, 2019; Burley et al., 2022).

#### 4.4.1 Personalized medicine

In personalized medicine, 3D organoids derived from ALS patient iPSCs can be used to test specific therapeutic approaches tailored to individual genetic and phenotypic profiles. These patient-specific organoids closely mimic the patient’s unique disease pathology, allowing researchers to evaluate the efficacy and safety of potential treatments in a more precise and personalized manner (Wichterle and Przedborski, 2010; Smits et al., 2019). For example, a study leveraged 3D spinal cord organoids derived from ALS patient iPSCs and electrophysiological tools to model disease-specific phenotypes and assess the efficacy of potential therapeutic compounds, identifying already-approved drugs with targets that modulate TDP-43 aggregates (Burkhardt et al., 2013). Additionally, studies utilizing motor neuron organoids derived from ALS patient iPSCs to conduct drug screening, concluding that patient-specific 3D organoid research, with the help of bioinformatics, has the potential to circumnavigate the limitations of ALS research and give rise to new drug and treatment discoveries (Amoros et al., 2022). This approach not only enhances the likelihood of therapeutic success but also minimizes adverse effects, leading to more effective and personalized healthcare solutions (Burley et al., 2022).

#### 4.4.2 Drug discovery and testing

3D organoids serve as powerful platforms for drug discovery and testing, providing a scalable and reproducible system for high-throughput screening of therapeutic compounds (Clevers, 2016). Organoids’ ability to mimic the complex cellular environment of human tissues allows for more accurate predictions of drug efficacy and toxicity (Fatehullah et al., 2016). In ALS research, organoids can be used to identify compounds that modulate neuronal excitability, reduce protein aggregation, or enhance cell survival. Additionally, organoid models can facilitate the discovery of novel biomarkers for disease progression and treatment response, further advancing the development of targeted therapies (Wainger et al., 2021).

#### 4.4.3 Transplantation of organoids

The transplantation of organoids offers significant potential in the treatment of ALS. Recent advancements have demonstrated that 3D organoids derived from human iPSCs can successfully integrate with tissue and contribute to functional recovery when implanted into animal models (Revah et al., 2022; Jgamadze et al., 2023). For instance, human-derived 3D cortical and spinal cord organoids formed synaptic connections and promoted neural regeneration when transplanted in mouse models with spinal cord injuries (Xu J. et al., 2023). However, several limitations related to vascularized structure formation, cell viability, and tissue integration remain before clinical applications of organoid transplantation can occur (Sagrac et al., 2021). Optimizing organoid transplantation involves integrating bioengineering technologies into the culturing process. For example, the vascularization of human brain organoids was shown to enhance the survival, integration, and functionality of transplanted organoids (Pham et al., 2018). To translate these organoids into clinical practice for treating patients with intractable diseases, challenges such as precise identification of the composition and size of cells forming the 3D structure, quality assurance, and mass production need to be addressed (Choi et al., 2023).

While 3D organoid models offer significant advantages in replicating human tissue complexity and cellular diversity, they also face limitations such as variability in organoid formation, lack of vascularization, and challenges in long-term culture (Huch and Koo, 2015; Lancaster et al., 2017). Standardization of organoid protocols and incorporation of vascular components are critical for improving the consistency and physiological relevance of these models (Pham et al., 2018). Future advancements should focus on integrating advanced imaging techniques, microfluidic systems, and bioengineering approaches to enhance the functionality and applicability of organoid models (Takebe et al., 2017). Additionally, combining organoids with other innovative technologies, such as single-cell RNA sequencing and CRISPR-based gene editing, will provide deeper insights into ALS pathogenesis and facilitate the development of more effective therapies (Sato et al., 2023).

## 5 Discussion

Electrophysiology has been essential in elucidating ALS mechanisms, revealing motor neuron dysfunction, ion channel changes, and network activity through techniques such as patch clamp, calcium imaging, and MEAs (Brette et al., 2008; Wainger et al., 2014; Yang et al., 2023). Complementary clinical techniques like MUNE and EIM further enrich our understanding by assessing motor neuron loss and muscle health (Shefner et al., 2011; Shefner et al., 2018). Recent advances allow electrophysiology techniques to be applied to organoids, bridging cellular-level insights with more complex, tissue-level analyses (Soscia et al., 2020).

The application of organoid research in the study of ALS pathology offers both promise and challenges (Chow et al., 2022). A critical challenge lies in the standardization and reproducibility of organoid protocols. Organoids differ in structure and activity patterns, and even organoids produced by the same iPS cell line may not be consistent. The existence of “batch syndrome,” where there is significant variability in both regional features and overall quality among produced organoids, poses a significant hindrance (Renner et al., 2017; Ilieva et al., 2018). Moreover, limitations in the progression and maturation of current brain organoid models during differentiation present another hurdle (Lancaster et al., 2013).

Human brain organoids take longer to produce specific cell types, such as upper-layer neurons and astrocytes, and often lack certain cell types and structures, such as the blood and immune systems. These might be minor concerns for organoid application, but the lack of a complete input/output system can be an important limitation in organoid research, although this can also be advantageous for dissecting intricate mechanisms in simplified systems. Another major constraint is the inability to stably observe and manipulate organoids over long periods, which hinders their application in ALS research. Conducting pathological research on organoids for extended periods, on a larger scale, and with more diverse methods remains a significant challenge for their effective use as ALS models.

Another challenge of organoid electrophysiology stems from current limitations in comprehensively probing structural, genetic, and functional properties, as well as heterogeneities across organoids over time. While bioelectronics enable long-term, stable recording of single-cell activities, there are constraints in specifically probing neuronal (or myofiber) activities with cell-type specificity and simultaneously recording from a large number of neurons within the 3D volume. Future advancements should prioritize developing technologies that enhance the analyses of organoid electrophysiological properties. This necessitates multimodal recording approaches integrating bioelectronics with *in situ* sequencing or cell-type-specific imaging to overcome existing limitations, allowing specific probing of neuronal activities and simultaneous recording from a larger neuron population within the 3D structure of brain organoids (Wang et al., 2018; Li et al., 2023).

One can envision applying some or ultimately all the electrophysiological techniques discussed here to organoids. For the more clinically relevant techniques, it would be relatively simple to perform EIM on the organoids. Indeed, there are impedance electrode-based cell culture dishes expressly for the purpose of measuring impedance of cells (Canali et al., 2015). Similarly, using an MEA would be possible in organoids using a similar concept (Tasnim and Liu, 2022). Performing other more clinically focused methods in organoids would depend on the complexity and organization of the organoid. For example, MUNE or MUNIX would require actual motor neurons and functioning synapses (Shin-Yi Lin et al., 2024). The same would also be true for NMJ assessments (Wang and Frank, 2024). More basic techniques such as patch clamp or calcium imaging, could be easily applied to organoids without significant challenges (Cakir et al., 2019; Sakaguchi et al., 2019). Additionally, integrating advanced imaging techniques and microfluidic systems into organoid studies could offer more detailed analyses and dynamic studies, respectively, thereby enhancing the physiological relevance of organoid models for understanding ALS pathology.

In conclusion, electrophysiological assessment of ALS, from single cells to humans, offers considerable potential value and remarkable flexibility. However, the application of these established techniques to organoid research in ALS will require a concerted effort to achieve standardization, reproducibility, and a more comprehensive representation of all cell types. Addressing issues such as batch variability, refining differentiation processes, and enhancing the ability to probe neuronal and myofiber activities within the 3D volume of tissue will significantly contribute to advancing our understanding of ALS pathology. These efforts pave the way for nuanced investigations into electrical signals, network-scale recordings, and excitability patterns, offering promising new avenues for assessing targeted therapeutic developments in ALS.
